# Activity Improvement and Vital Amino Acid Identification on the Marine-Derived Quorum Quenching Enzyme MomL by Protein Engineering

**DOI:** 10.3390/md17050300

**Published:** 2019-05-21

**Authors:** Jiayi Wang, Jing Lin, Yunhui Zhang, Jingjing Zhang, Tao Feng, Hui Li, Xianghong Wang, Qingyang Sun, Xiaohua Zhang, Yan Wang

**Affiliations:** 1 College of Marine Life Sciences, Ocean University of China, Qingdao 266003, China; wangjiayi109911@163.com (J.W.); lynn44944@163.com (J.L.); yhzhang2011@163.com (Y.Z.); jingjingzhangnn@163.com (J.Z.); fengtao246@163.com (T.F.); l56021831@163.com (H.L.); xhwang@ouc.edu.cn (X.W.); lilysun1012@126.com (Q.S.); xhzhang@ouc.edu.cn (X.Z.); 2 Laboratory for Marine Ecology and Environmental Science, Qingdao National Laboratory for Marine Science and Technology, Qingdao 266071, China; 3 Institute of Evolution & Marine Biodiversity, Ocean University of China, Qingdao 266003, China

**Keywords:** quorum quenching enzyme, error prone PCR, high-throughput screening, site-directed mutagenesis, catalytic ability, *Pectobacterium carotovorum* subsp. *carotovorum (Pcc)*

## Abstract

MomL is a marine-derived quorum-quenching (QQ) lactonase which can degrade various *N*-acyl homoserine lactones (AHLs). Intentional modification of MomL may lead to a highly efficient QQ enzyme with broad application potential. In this study, we used a rapid and efficient method combining error-prone polymerase chain reaction (epPCR), high-throughput screening and site-directed mutagenesis to identify highly active MomL mutants. In this way, we obtained two candidate mutants, MomL_I144V_ and MomL_V149A_. These two mutants exhibited enhanced activities and blocked the production of pathogenic factors of *Pectobacterium carotovorum* subsp. *carotovorum (Pcc)*. Besides, seven amino acids which are vital for MomL enzyme activity were identified. Substitutions of these amino acids (E238G/K205E/L254R) in MomL led to almost complete loss of its QQ activity. We then tested the effect of MomL and its mutants on *Pcc*-infected Chinese cabbage. The results indicated that MomL and its mutants (MomL_L254R_, MomL_I144V_, MomL_V149A_) significantly decreased the pathogenicity of *Pcc*. This study provides an efficient method for QQ enzyme modification and gives us new clues for further investigation on the catalytic mechanism of QQ lactonase.

## 1. Introduction

Quorum sensing (QS) is a communication system that many bacteria aggregates used to regulate their aggregate size via small molecules called autoinducers [1,2]. The process of interfering with QS through degradation of signals is termed as quorum quenching (QQ). *N*-acyl homoserine lactones (AHLs) are QS signals used by a wide range of Gram-negative bacteria. AHL lactonase is one major type of AHL-degrading enzymes, which hydrolyses the lactone ring of AHL molecule to produce corresponding *N*-acyl-homoserine. Since some bacteria use QS to mediate virulence factors and antimicrobial resistance, QQ is considered to be a promising alternative for bacterial disease control, which can attenuate QS-regulated virulence factors production in many bacterial pathogens without any lethal effect and impart less-selective pressures for resistant mutants than conventional antibiotics [3,4,5]. QQ enzyme is one of the most well-studied methods of QQ. AiiA, the earliest identified QQ enzyme, can decrease extracellular pectolytic enzyme activity and attenuate pathogenicity of *Erwinia carotovora* [6]. A recent study shows that QQ enzyme (AiiA) and QS inhibitor (G1) demonstrated enhanced QS inhibiting effects on reducing AHL concentration when applied together [7]. 

MomL, a novel AHL lactonase, was isolated from *Muricauda olearia* Th120 [8,9]. This protein consists of 294 amino acids and has a molecular weight of 32.8 kDa. MomL belongs to the metallo-β-lactamase superfamily, and shows the highest identity of 56.8% with protein Aii20J, which belongs to *Tenacibaculum* sp. 20J [10]. Moreover, MomL shares 54.4% and 24.5% identity with FiaL from *Flaviramulus ichthyoenteri* T78T and AiiA from *Bacillus* sp. 240B1 [10,11,12,13,14]. The wide-ranging substrate properties of MomL confer great advantages in disease prevention because different pathogenic bacteria produce AHL molecules with different chain lengths. For example, AHL produced by *Burkholderia* is C8-HSL [15,16,17], while that of *Vibrio harveyi* is 3OC4-HSL [18]. Moreover, the ability of MomL to degrade C6-HSL is approximately 10 times higher than that of AiiA [14]. MomL exhibited degradative activity on both short and long-chain AHLs and inhibited the pathogenicity of different pathogenic bacteria [9,19]. In order to investigate its application value, MomL was heterologously expressed by *Bacillus brevis*, and the recombinant strain showed a broad antibacterial spectrum than original strain [20]. Although MomL shares the “HXHXDH~H~D” motif with other AHL lactonases in the metallo-β-lactamase superfamily, this motif of MomL performs different functions from AiiA [14,21,22]. Furthermore, little is known about its catalytic mechanism and other amino acids that are involved in the active site remain unclear. Therefore, elucidating the action mechanism helps to expand the application of MomL and paves a way for marine-derived QQ enzyme research. 

*Pectobacterium carotovorum* subsp. *carotovorum (Pcc)* is a bacterial pathogen that can cause severe soft rot of cabbage [23,24,25]. Extracellular enzymes such as pectate lyases, pectinases, cellulases and proteases produced by *Pcc* are main causes for tissue maceration [26]. Disease factors produced by *Pcc* can be induced by the AHL-based QS system [27]. Thus, as an environmentally friendly biocontrol strategy, QQ can be used to prevent or alleviate symptoms caused by such infections.

Protein engineering is a multi-faceted field that can create desired protein properties via various approaches including protein structure prediction to protein selection from random mutagenesis library [28]. As an early example, the *ebgA* gene of *E. coli K12*, was deleted to lead to the synthesis of ebg enzyme and show enhanced activity toward lactose [29]. The catalytic function of cytochrome c from *Rhodothermus marinus* was enhanced more than 15-fold than industrial catalysts in forming carbon-silicon bonds [30,31]. Building high-quality mutant libraries and high efficiency screening system are crucial steps for selecting functional proteins. Site-directed mutagenesis is a valuable tool for understanding the relationship between enzyme activity and amino acids. 

In this study, we improved the efficiency of mutant library establishment using a combination method of error-prone polymerase chain reaction (epPCR) and seamless cloning. In addition, an IPTG in situ photocopying technology was used to perform high-throughput screening of random mutagenesis library. We rapidly obtained two high-activity mutant proteins and identified seven amino acids which are vital for QQ ability of MomL. Furthermore, we investigated the ability of MomL and its mutants to inhibit the agricultural pathogenic bacterium *Pcc* virulence factors and the formation of soft rot on Chinese cabbage.

## 2. Results 

### 2.1. Overview of the High-Efficiency Strategy of Constructing and Screening a Random Mutagenesis Library 

**Figure 1:**
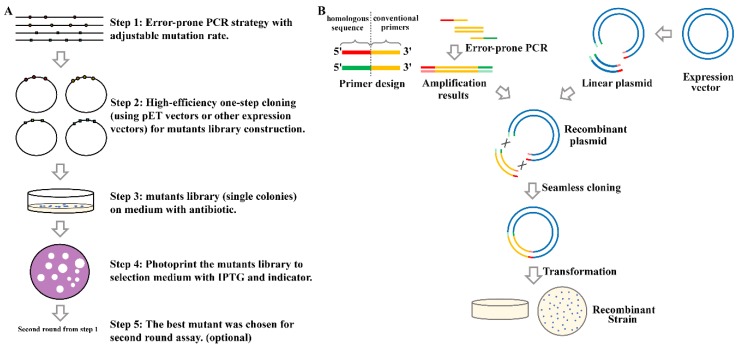
The schematic diagram of high efficiency strategy of constructing and screening random mutagenesis library (**A**) and the process of error-prone polymerase chain reaction (epPCR) and seamless cloning (**B**).

In this study, we built a highly efficient and rapid method to obtain the required variants. This method mainly combined three types of technology, specifically epPCR, seamless cloning and isopropyl-β-d-thiogalactoside (IPTG) in situ photocopying. We selected an appropriate amino acid mutation rate and generated PCR products containing randomly mutated amino acids by performing optimized epPCR of three rounds. The PCR products were cloned into pET-24a(+) vectors via seamless cloning, and the recombinant plasmids were transformed into *E. coli* BL21(DE3). *Chromobacterium violaceum* CV026 can produce violacein in the presence of AHLs with *N*-acyl side chains from C4 to C8 in length. When QQ substances were added, the production of violacein was inhibited. Therefore, in the screening plate containing exogenous C6-HSL and the indicator CV026, C6-HSL can be degraded and the plate will not turn violet when the imprinted *E. coli* BL21 colonies of the random mutagenesis library produced active MomL enzyme. Single colonies were imprinted on the screening plates containing IPTG and indicator CV026. The QQ ability of MomL was estimated by either the white halo or the halo diameter produced in the screening plate and positive mutants were selected. The method used in this study was highly efficient and faster than the traditional method (Figure 1). The analyzation for the efficiency and feasibility of this method were performed using MomL protein as an example.

### 2.2. Error-Prone Polymerase Chain Reaction (EpPCR) Condition Optimization with Suitable Mutation Efficiency

EpPCR randomly introduces mutant sites, and the mismatch rate is related to the magnesium and manganese ion contents [32,33]. In order to build a more efficient mutant library, 1% were selected as the optimal amino acid mutation rate. To determine the appropriate mismatch rate, Mg^2+^ concentration gradient ranging from 1 to 8 mM and Mn^2+^ gradient ranging from 0 to 0.6 mM were detected respectively. As shown in Figure S1A,B, specific DNA bands were observed following PCR in different Mg^2+^ or Mn^2+^ concentration gradient. Next, orthogonal test of the two factors (Mg^2+^ and Mn^2+^) was conducted based on the results of the single factor experiment. Appropriate DNA bands were obtained under the 10 orthogonal test conditions (Figure S1C). We randomly selected 100 single colonies of each condition for sequencing. The results demonstrated that under 1 mM Mg^2+^ and 0.2 mM Mn^2+^ conditions, 70% of single colonies were suffered in 1–3 mutant sites while no mutation was presented in the other 23% of colonies. Fifty percent of the mutant proteins contained no more than 2 mutant sites under the 2 mM Mg^2+^ and 0.1 mM Mn^2+^ conditions. The average mutation frequency distribution was under 2 mM Mg^2+^ and 0.15 mM Mn^2+^, including 2-3 mutant sites in 50% of mutant proteins and 2-5 mutant sites in 83% of mutant proteins. Besides, under this condition, 100 detected mutant libraries existed at least one mutant site. Under 2 mM Mg^2+^ and 0.2 mM Mn^2+^, premature translational termination occurred in 30% of mutant proteins, and there were 7–11 mutant sites per protein. Ultimately, based on these results, 2 mM Mg^2+^ and 0.15 mM Mn^2+^ conditions were selected to maintain the amino acid mutation rate at approximately 1% (Table S2).

### 2.3. Screening of a Mutation Library Based on a Seamless and EpPCR Strategy

**Figure 2:**
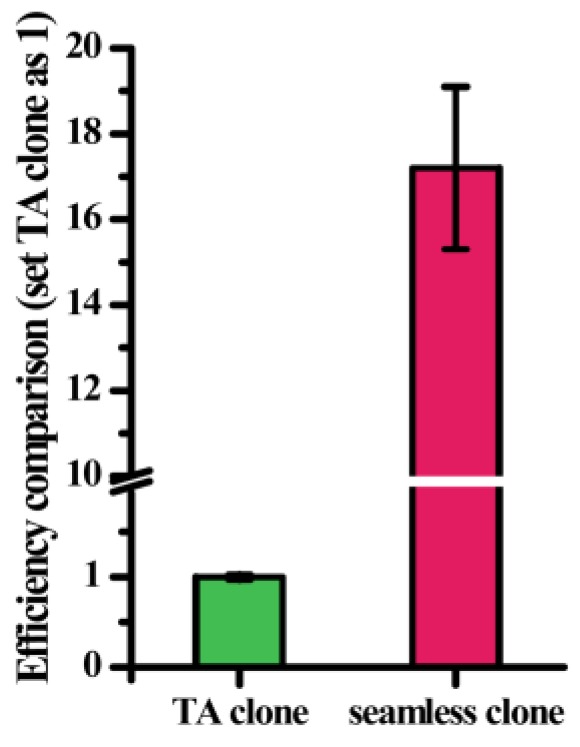
Efficiency comparison between seamless cloning and traditional cloning (TA). All data are presented as mean ± standard deviation (SD, *n* = 3).

A random mutagenesis library was built using the selected epPCR conditions. Next, the traditional cloning method (TA cloning) and seamless cloning were separately employed to ligate random mutant fragments and vectors. There were obvious differences in mutation library abundance between the two methods. The number of mutant proteins obtained via seamless cloning was 15–20 times higher than that obtained using TA cloning (Figure 2). Besides, randomly sequencing results showed that 92% of single colonies contained the *momL* gene while only 8% were false positive colonies with self-ligation plasmids. 

**Figure 3:**
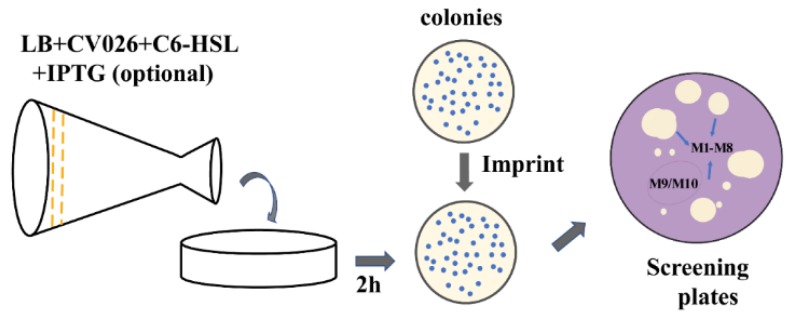
Screening target proteins by isopropyl-β-d-thiogalactoside (IPTG) *in situ* photocopying. M1–M8 are single colonies with highly activity; M9–M10 indicate inactive proteins.

We obtained more than 5000 mutant strains for random mutagenesis library. Subsequently, IPTG in situ photocopying technology was utilized to efficiently screen mutant proteins. In the prescreening step, QQ ability of MomL was estimated by whether the visual white halo showed in screening plate. In this step, approximately 3000 strains were screened; 10% of strains that produced larger halo diameters were chosen for second-round screening. In second round screening step, mutants were screened using crude enzyme supernatant in CV026-loaded screening plate. Single colonies M1–M8 were selected from the area with large white halos while M9–M10 were identified from the region lacking white halos (Figure 3 and Figure S2). Two high-activity mutant proteins, M2 and M3, and the mutant proteins M9 and M10, which lacked activity, were selected for sequencing. The results indicated that Ile144 in M2 was mutated to Val (I144V), and Val149 in M3 was mutated to Ala (V149A). In addition, four amino acids in M9 were mutated, namely, E238G, N179S, N51Y and K82R, and four amino acids in M10 were mutated, namely, M228V, T84A, K205E and L254R. 

### 2.4. Analysis of Amino Acids in Mutant Proteins

**Figure 4:**
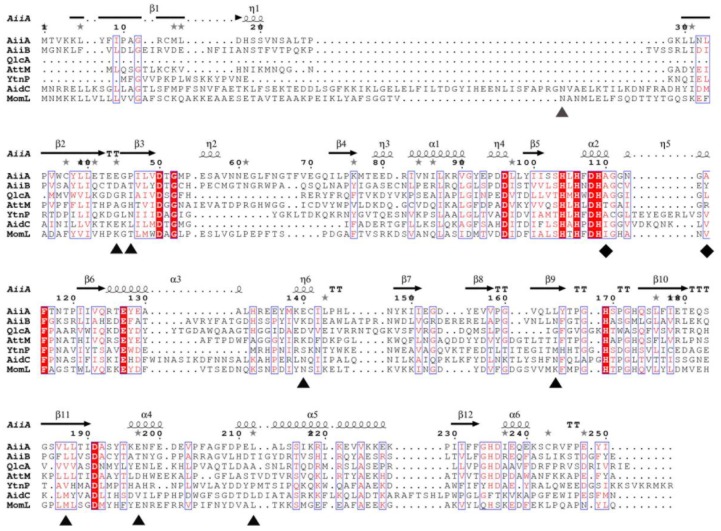
Multiple sequence alignment of amino acid sequences of MomL, putative homologues, and other representative *N*-acyl homoserine lactone (AHL) lactonases. Sequence alignment was performed by the MUSCLE program in the MEGA software package and enhanced by ESPript 3.0. MomL homologue from *Eudoraea adriatica* (WP_019670967) showed the highest score when BLASTP searching nonredundant (NR) databases. Other sequences of AHL lactonase are AiiA from *Bacillus* sp. strain 240B1 (AAF62398), AidC from *Chryseobacterium* sp. strain StRB126 (BAM28988), QlcA from unculturable soil bacteria, and AttM (AAD43990), AiiB (NP 396590) from *Agrobacterium fabrum* C58 and YtnP from *Bacillus*. Filled triangles show amino acids which are essential for MomL activity. Filled rhombuses show amino acids, the mutation of which increased MomL activity.

**Figure 5:**
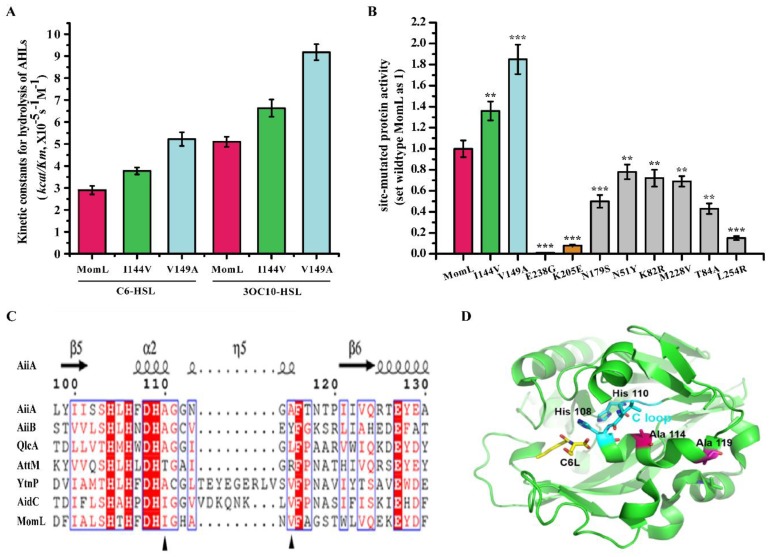
(**A**) Enzyme kinetics experiments of MomL and mutant proteins on different substrates C6-HSL and 3OC10-HSL. (**B**) Protein activity test of mutant proteins. All data are presented as mean ± standard deviation (SD, *n* = 3). An unpaired t-test was performed for testing significant differences between groups (*** *P* < 0.001, ** *P* < 0.01, * *P* < 0.05). (**C**) Multiple-sequence alignment of the amino acid sequences of MomL, putative homologues, and other representative AHL lactonases. The multiple-sequence alignment procedure is the same as described in Figure 4. (**D**) The structure and active site of AiiA, the homologous protein of MomL. A114 and A119 in AiiA are located near the C-loop.

To further analyze the functions of single amino acids, we mutated the above amino acids loci and constructed 10 single amino acid mutants: MomL_I144V_, MomL_V149A_, MomL_N51Y_, MomL_N179S_, MomL_M228V_, MomL_K205E_, MomL_E238G_, MomL_L254R_, MomL_T84A_, and MomL_K82R_ (Figure 4). Biochemical test indicated that the activities of MomL_I144V_ and MomL_V149A_ were 1.3 and 1.8 times higher, respectively, than that of wild-type MomL (Figure 5A). Furthermore, MomL_E238G_ was inactive, and the activities of MomL_K205E_ and MomL_L254R_ were reduced by 80%–90% compared to MomL. The activities of MomL_N179S_/MomL_N51Y_/MomL_K82R_/MomL_M228V_/MomL_T84A_ also decreased, ranging from 40–80% of wild-type MomL activity (Figure 5B). The results indicated that Glu238, Lys205, Leu254, Thr84 and Asn179 are related to hydrolysis reaction of C6-HSL. Changes in every single site can reduce the enzyme activity to 50% or more. 

By screening mutant proteins, we rapidly obtained two live mutant proteins and identified seven amino acids that are involved in QQ ability of MomL. By multiple sequence alignment of MomL and other AHL lactonases belonging to the metallo-β-lactonase superfamily, we found that Ile144, Val149, Asn179, Lys205 were variable amino acids in the conserved domain “HXHXDH ~ 60aa ~ H”, and may be directly related to the catalytic reaction; while Thr84, Glu238 and Leu254 were amino acids outside the conserved domain, and may be related to maintaining protein stability. In addition, by analyzing the structure of AiiA, the homologous protein of MomL, we found that I144 and V149 in MomL (A114 and A119 in AiiA) are located near the catalytic ring of the active center (C-loop in Figure 5C,D). We speculated that the mutation of I144V and V149A may affect the enzyme activity by affecting the conformation of the C-loop.

### 2.5. The Effect of Mutant Proteins on the Virulence Factors and Survival of Pectobacterium Carotovorum *Subsp.* Carotovorum (Pcc)

**Figure 6:**
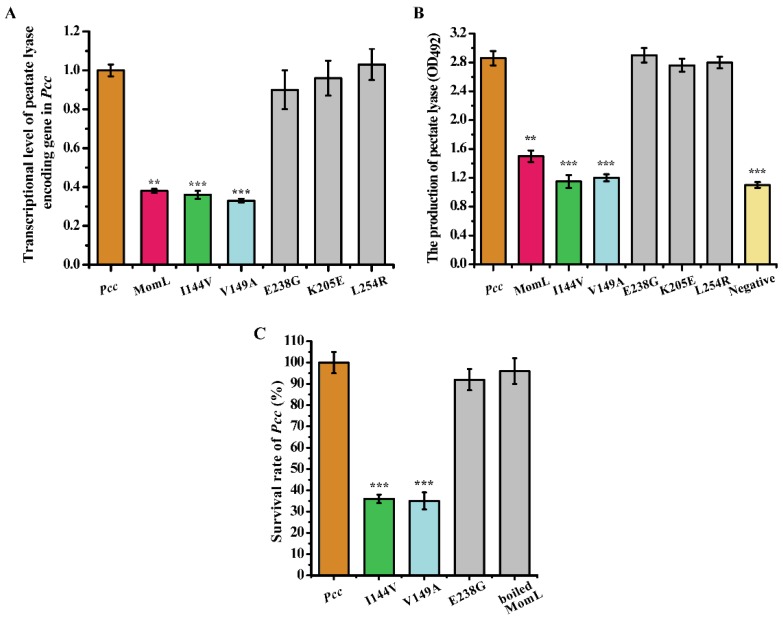
Transcriptional level of pectate lyase encoding gene in *Pectobacterium carotovorum* subsp. *carotovorum* (*Pcc*) (**A**) and the production of pectate lyase (OD_492_). (**B**). Effects of MomL and mutant proteins towards the *Pcc* survival rate (**C**). All data are presented as mean ± standard deviation (SD, *n* = 3). An unpaired t-test was performed for testing significant differences between groups (*** *P* < 0.001, ** *P* < 0.01, * *P* < 0.05).

The inhibitory effects of mutant proteins on pectate lyase, the virulence factor of the plant pathogenic bacterium *Pcc*, was analyzed. Wild-type MomL, MomL_I144V_ and MomL_V149A_ inhibited the expression of the pectate lyase gene, and the inhibitory effect of MomL_V149A_ was slightly higher than the wild-type MomL. We also analyzed the gene expression of pectate lyase when treated by MomL_E238G_, MomL_K205E_ and MomL_L254R_. The mutation of these three amino acids resulted in the inability to inhibit pectate lyase gene expression (Figure 6A). In addition, the yield of pectate lyase was determined and the results were consistent with the transcriptional analysis. MomL_I144V_ and MomL_V149A_ greatly reduced pectate lyase yield, while MomL_E238G_, MomL_K205E_ and MomL_L254R_ did not (Figure 6B). Besides, the presence of MomL_I144V_ and MomL_V149A_ reduced the *Pcc* survival rate under stress conditions to 30%–45% of the survival of *Pcc* alone. The presence of MomL_E238G_ did not affect *Pcc* survival. Furthermore, the boiled MomL did not affect the *Pcc* survival rate (Figure 6C). We speculated that site-directed mutagenesis of *momL* led to changes in other function of the mutant proteins, such as the fold of the enzyme, stability, substrate interaction and many other performance parameters, and thus resulted in reduced survival of *Pcc*. However, the specific mechanism needs to be studied further. 

### 2.6. Effects of MomL and Mutant Proteins on Pcc Infection of Chinese Cabbage

**Figure 7:**
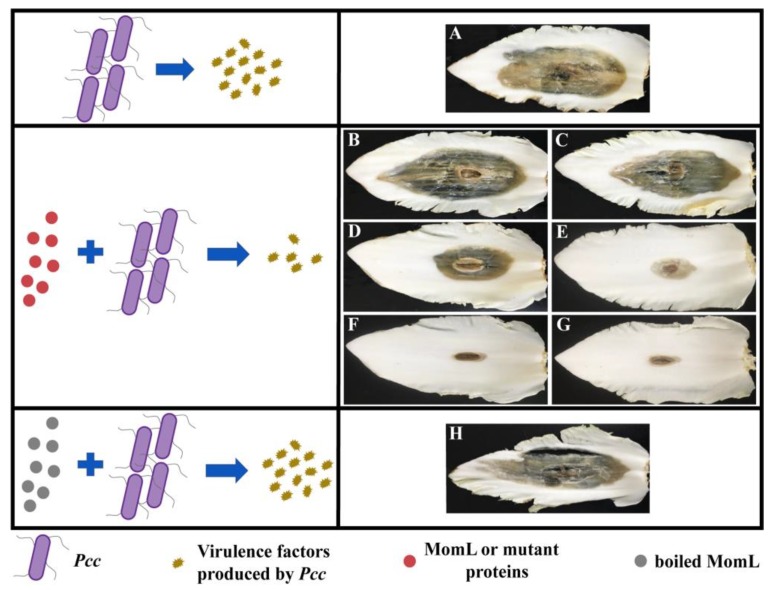
Effects of MomL and mutant proteins on *Pcc* infection of Chinese cabbage. (**A**) *Pcc*; (**B**) *Pcc* with MomL_E238G_; (**C**) *Pcc* with MomL_K205E_; (**D**) *Pcc* with MomL_L254R_; (**E**) *Pcc* with MomL; (**F**) *Pcc* with MomL_I144V_; (**G**) *Pcc* with MomL_V149A_; (**H**) *Pcc* with boiled MomL. The results shown are representative of biological duplicates.

To further analyze MomL and its mutants, their treatment effect towards soft rot of Chinese cabbage was tested. When treated by *Pcc* alone, approximately 2/3 of the cabbage leaf area was infected and decomposed. Following infection with *Pcc* and treatment with MomL, only a small percentage of tissue was infected. However, inactivated MomL applied in combination with *Pcc* did not reduce the degree of decay in Chinese cabbage. The decay areas of the cabbage after treatment with MomL_E238G_, MomL_K205E_ and *Pcc* were comparable to those obtained with *Pcc* infection alone. The application of MomL_L254R_ and *Pcc* together reduced the decay area by approximately 50% compared with that treated by *Pcc* alone. The treatment effects of MomL_I144V_ and MomL_V149A_ were the most significant. After the application of MomL_I144V_ and MomL_V149A_, the *Pcc* infection rate on Chinese cabbage decreased obviously (Figure 7). Overall, in infection experiments, the bacterial survival rate significantly decreased by more than 50% after adding MomL or active mutant proteins. The results indicated that co-culture with MomL or mutant proteins can relieve the symptoms caused by *Pcc*, and this may be due to the decrease of virulence factors such as pectate lyase. 

## 3. Discussion

Marine metagenomic data revealed that QQ is a common activity in marine bacteria [34]. Many QQ enzymes have been identified from marine species, such as Aii20J from *Tenacibaculum* sp. strain 20J, QsdH from *Pseudoalteromonas byunsanensis* strain 1A01261 and AiiC from *Anabaena* sp. PCC 7120 [35,36]. QQ enzymes have broad application prospects in aquaculture disease control, biofouling prevention and drugs development [37,38]. Improving the degrading ability of QQ enzymes will lead to highly stable and efficient proteins for industrial use. Thus, further studies about marine aquatic QQ can expand marine QQ bioresource application and pave a way to solve problems related to aquaculture and agriculture that is conducted in a saline environment [39]. The marine-derived QQ enzyme MomL, a novel type of AHL lactonase with an unknown action mechanism, was investigated in this study. MomL demonstrates a wide antimicrobial spectrum and provides a promising alternative for disease control due to its ability to inhibit the pathogenicity induced by the AHL QS system. The amino acids and active site in MomL have not previously been explored, except for the “HXHXDH~H~D” motif. Hence, we focused on MomL to improve its bacteriostatic activity, explore its highly active mutant proteins, and identify amino acids involved in enzyme activity via site-directed mutagenesis, thus providing a theoretical basis for its mechanism of action.

Among protein engineering strategies, random mutagenesis methods are usually applied to study properties that are not understood rationally. EpPCR is standard method for random mutagenesis due to its robustness and simplicity in use [40]. A seamless cloning technique is used to insert a targeted fragment into any location in the vector without relying on an enzymatic site. The main factor affecting epPCR was the concentration of Mn^2+^, which can result in higher mutation frequencies at higher concentrations. Other influential factors including the concentration of Mg^2+^, the proportion of deoxyribonucleoside triphosphates (dNTPs), and even the PCR reaction cycles. In this study, mutation frequencies were controlled at 1–3%. Thus, each protein contained 3-5 mutations. After multiple analyses, we ultimately determined the concentrations of dNTP, Mg^2+^ and Mn^2+^ for the use in next step. We screened amplification enzymes using epPCR to identify high-fidelity enzymes with improved cloning efficiency, but unsatisfactory mutation rates resulted in the low diversity of the random mutant library. Ultimately, Taq enzyme was chosen for epPCR. At the beginning of each reaction, the Taq enzyme produced higher mutant library diversity with 2–5 mutations per protein but achieved low seamless cloning efficiency, thus limiting the number of transformants. Presumably, the A-end of the Taq enzyme affected the efficiency of seamless cloning, which was optimized in our study. We removed the A-end using the HS DNA polymerase (Takara Primer STAR^®^). The entire experimental time was shortened to one-fifth of the time required for traditional experiment, and the efficiency of mutant library establishment was nearly 10 times higher than that achieved previously. Furthermore, the efficiency of positive cloning during mutant library construction was as high as 92%. Thus, our strategy demonstrated wide applications for establishing protein mutant libraries, and greatly improved the efficiency of seamless cloning.

The first two approaches involve large high-throughput selection, and only 10%–20% of bacteria on a parent plate can be transferred to a sub-plate in the traditional method. But IPTG in situ photocopying is a high-throughput screening system. By performing single colony dilution and counting the number of single colonies, we increased the transferred number of bacteria to 50%. This type of screening method holds great applicable value for other QQ enzymes’ screening. By screening mutant proteins, we rapidly obtained two highly active mutants of MomL and identified seven amino acids which are involved in enzyme activity. However, given the lack of MomL crystals structure, the deep catalytic mechanism remains to be characterized. In infection experiments, the bacterial survival rate significantly decreased by more than 50% after adding highly active mutant proteins to *Pcc*. MomL and its mutant proteins also reduced the virulence factor pectate lyase produced by *Pcc*. We applied these proteins to infect Chinese cabbage and found that the infection symptoms were alleviated after adding MomL or its mutants, indicating MomL and its mutants can be an alternative strategy for disease control. We are currently characterizing the minimum concentration and maximum time required for MomL treatment to facilitate the application of MomL alike to actual utilization.

## 4. Materials and Methods 

### 4.1. Bacterial Strains, Plasmids, Media, Growth Conditions, and Chemicals

*Pectobacterium carotovorum* subsp. *carotovorum* (*Pcc*) was purchased from the CGMCC (China General Microbiological Culture Collection, Beijing, China) [41]. *E. coli* strain AHL882-5 was used to express the MomL protein. *E. coli* strain BL21(DE3) was used as a host for protein expression. Proteins were expressed following the cloning of random mutants of the *momL* gene into pET-24a(+). The strain *Chromobacterium violaceum* CV026 was used as an indicator in the AHL activity bioassay [42]. C6-HSL was purchased from the Cayman Chemical Company and prepared in dimethyl sulfoxide (DMSO). *M. olearia* Th120, CV026 and *Pcc* were routinely cultured on Luria-Bertani (LB) agar at 28 °C. *E. coli* strain AHL882-5 was cultured in LB medium at 37 °C. When required, 25 μg/mL kanamycin was added to the solid or liquid media. 

### 4.2. Random Mutant Library Construction and Identification of High-Activity Mutants 

The mutant library of the AHL lactonase MomL was constructed using error prone PCR (epPCR). The primers for epPCR are listed in Table S1. Each 100-μL epPCR reaction contained 10 μL of 10× PCR buffer (Takara, Shiga, Japan), 8 μL of dNTP mixture (2.5 mM dATP, 2.5 mM dGTP, 10 mM dCTP, and 10 mM dTTP), 1 μL of the primer *momL*-F (20 μM), 1 μL of the primer *momL*-R (20 μM), 1 μL of template plasmid from strain AHL882-5, 1 μL of Taq DNA Polymerase (Takara, 5 U/μL), appropriate metal ions, and deionized water to a final volume of 100 μL. PCR was conducted using the following conditions: denaturation at 94 °C for 10 min, followed by 30 cycles of denaturation at 94 °C for 30 s, annealing at 55 °C for 30 s, extension at 72 °C for 60 s, and a final incubation at 72 °C for 10 min. The resulting PCR products were digested with Prime STAR^®^ HS DNA Polymerase (Takara) to improve the ligation efficiency. They were then further digested with DpnI (NEB, Ipswich, MA, USA) to remove template plasmids and were finally purified using a PCR product purification kit (Biomed, Beijing, China) according to the manufacturer’s instructions. Purified mutant *momL* genes were ligated into the linear vector pET-24a(+) via seamless cloning. Recombinant plasmids were transformed into *E. coli* BL21(DE3), diluted with fresh LB medium, plated on LB agar containing 25 μg/mL kanamycin, and cultured at 37 °C overnight.

### 4.3. High-Throughput Screening of High-Activity Mutants

We added 1 mL of overnight cultured CV026, 7.5 µl C6-HSL (DMSO, 1 mM) and 0.5 mM IPTG (final concentration) to 15 mL of molten semisolid LB agar (1%, *w*/*v*) before the agar was poured into the plates. When the agar solidified, colonies growing on LB agar containing 25 μg/mL kanamycin were imprinted on the selection plate using sterile toothpicks. After the prescreening step, choosing mutants that produced a white halo for the next round screening. In second-round screening, the mutants were induced to expression in 0.5 mM IPTG condition, the supernatant of the cultures were collected after centrifugation at 12,000 rpm for 10 min at 4 °C and filtered through 0.22-μm-pore-size filter to test the AHL lactonase activity. The CV026 screening plate was prepared as described above without adding with 0.5 mM IPTG. The medium was punched using a sterile tip and the crude enzyme supernatant were added into the hole (with MomL crude enzyme supernatant as positive control and LB medium as negative control).

### 4.4. Expression and Purification of Mutant Proteins 

Colonies surrounded by white halos on the purple background of the plate were picked and cultured in LB medium (with 25 μg/mL kanamycin) in a shaking incubator at 37 °C. Protein expression was induced with 0.5 mM IPTG at an original OD_600_ of 0.5–0.7 at 16 °C for 12 h. Cells were harvested via centrifugation at 12,000 rpm for 10 min at 4 °C. Cell pellets were resuspended gently in binding buffer (20 mM Tris-HCl with 10 mM imidazole, 0.5 M NaCl, pH 8) and disrupted by sonication on ice. The cell debris was removed via centrifugation at 12,000 rpm for 10 min at 4 °C, and the supernatants were filtered through a 0.22 μm pore-size filter. Before they were loaded onto NTA-Ni (Qiagen) columns, the supernatants were confirmed using a CV026 plate assay previously described by McClean [42]. Then, the mutant proteins were eluted using a specific wash buffer (20 mM Tris-HCl containing different amounts of imidazole, 0.5 M NaCl, pH 8) from the NTA-Ni columns and evaluated by sodium dodecyl sulfate polyacrylamide gel electrophoresis (SDS-PAGE).

### 4.5. N-Acyl Homoserine Lactone (AHL) Lactonases Activity Assay 

The relative activity of mutant proteins was measured using a pH-sensitive colorimetric assay previously described by K Tang [9]. The test system consisted of a reaction buffer, morpholinepropanesulfonic acid (MOPS), a pH indicator, bromothymol blue (BTB), AHL substrate C6-HSL and the enzyme undergoing measurement. When AHL molecules were degraded to donate protons, the pH was weakly altered. Color changes due to BTB were measured using a microplate reader.

### 4.6. Site-Directed Mutagenesis of Moml

To study multi-site mutant proteins, we constructed site-directed single mutant via site-directed mutagenesis [43]. Mutation sites and primers [44] are listed in Table S1. The mutated genes were amplified using Primer STAR GXL DNA polymerase (Takara) and cyclized after phosphorylation. Recombinant plasmids were expressed in *E. coli* BL21(DE3) and mutant proteins were purified as previously described. The enzyme activity of each mutant protein was measured as described above.

### 4.7. Kinetic Assay of Moml and Mutant Proteins Activities

The catalytic activities of MomL and mutant proteins were measured by a pH sensitive colorimetric assay [45]. Briefly, 3.5nM enzyme, C6-HSL/3OC10-HSL (0.156 to 5 mM) were added to a MOPS (5 mM, pH 7.1)/BTB (1 mM) system of total 100 uL. Due to the pH-sensitive dye (BTB) mediated color change, OD_630_ was continuously measured using a microplate absorbance reader [9]. Initial rates were calculated and a GraphPad Prism software was used for calculating *K_m_* and *k*_cat_ values. A standard curve using HCl was constructed to reflect the relationship between the absorbance change and the proton concentration, the value of OD_630_ would decrease by 0.193 after adding with 100 nmol of HCl. 

### 4.8. Effects of MomL and Mutant Proteins on the Pathogenicity of Pcc

*Pcc* was cultured to the exponential phase and inoculated in 5 mL of LB medium containing equal amounts of enzymes (MomL, MomL_I144V_ and MomL_V149A_), and ddH_2_O was added as a positive control. The cultures were grown on a shaker (170 rpm) at 28 °C for 24 h. Extracellular pectate lyase activity was determined using a DNS assay. First, 0.2% polygalacturonic acid reaction buffer (0.2% polygalacturonic acid; 0.2 M NaCl in 0.05 M sodium acetate buffer at pH 5.2) was prepared. Then, to obtain culture samples for enzyme assays, cultures were centrifuged at 4000 rpm for 5 min, and the supernatants were filter-sterilized through a 0.22 µm filter at 4 °C or on ice. Two hundred microliters of enzyme and 400 μL of 0.2% polygalacturonic acid reaction buffer were blended and immersed in a tube maintained at a constant temperature of 48 °C for 30 min. Four hundred microliters of DNS were added to the 600 μL reaction system, and the mixture was incubated in boiling water for 5 min and centrifuged at 12,000 rpm for 1 min; the precipitate was then discarded. The supernatant was diluted three times, and the absorbance was measured at 492 nm. 

### 4.9. Pcc Survival Rate Assay

*Pcc* was cultured as mentioned above. A bacterial suspension was diluted with fresh LB and plated on LB agar; after 15 h of growth at 28 °C, colonies were counted. The colonies on *Pcc* plates were set to 100%.

### 4.10. Pcc Infection Experiment

Leaves were selected from the same Chinese cabbage, and 10 μL of *Pcc* bacterial suspension with enzyme (MomL and mutant proteins) were added to a cut surface. The inoculated leaves were incubated in sterile dishes at 28 °C for 24–48 h. *Pcc* alone was used as the positive control.

## Supplementary Materials

The following are available online at https://www.mdpi.com/1660-3397/17/5/300/s1. Figure S1: The detection of gel electrophoresis of *momL* fragment with series of epPCR condition. Figure S2: The protein activity test. Table S1: Mutation sites and mutation primers of MomL. Table S2: The mutation rate comparison of epPCR products with different conditions.
