# Supplementary material for: Activity Improvement and Vital Amino Acid Identification on the Marine-Derived Quorum Quenching Enzyme MomL by Protein Engineering
Source: Mar Drugs. 2019 May 21;17(5):300. doi: 10.3390/md17050300 (PMC6562636; doi:10.3390/md17050300)
Supplement: Supplementary file 1 [file marinedrugs-17-00300-s001.pdf]

# Activity improvement and vital amino acid identification on the marine-derived quorum quenching enzyme MomL by protein engineering

**Jiayi Wang [1,†], Jing Lin [1,†], Yunhui Zhang [1], Jingjing Zhang [1], Tao Feng [1], Hui Li [1], Xianghong Wang [1], Qingyang Sun [1], Xiaohua Zhang [1,2,3] and Yan Wang [1,2,3*]**

[1] College of Marine Life Sciences, Ocean University of China, Qingdao, 266003, China; wangjiayi109911@163.com (J.W.); lynn44944@163.com (J.L.); yhzhang2011@163.com (Y.Z.); jingjingzhangnn@163.com (J.Z.); fengtao246@163.com (T.F.); l56021831@163.com (H.L.); xhwang@ouc.edu.cn (X.W.); lilysun1012@126.com (Q.S.); xhzhang@ouc.edu.cn (X.Z.)

[2] Laboratory for Marine Ecology and Environmental Science, Qingdao National Laboratory for Marine Science and Technology, Qingdao 266071, China

[3] Institute of Evolution & Marine Biodiversity, Ocean University of China, Qingdao, 266003, China

[*] Correspondence: wangy12@ouc.edu.cn

† These authors contributed equally to this work.

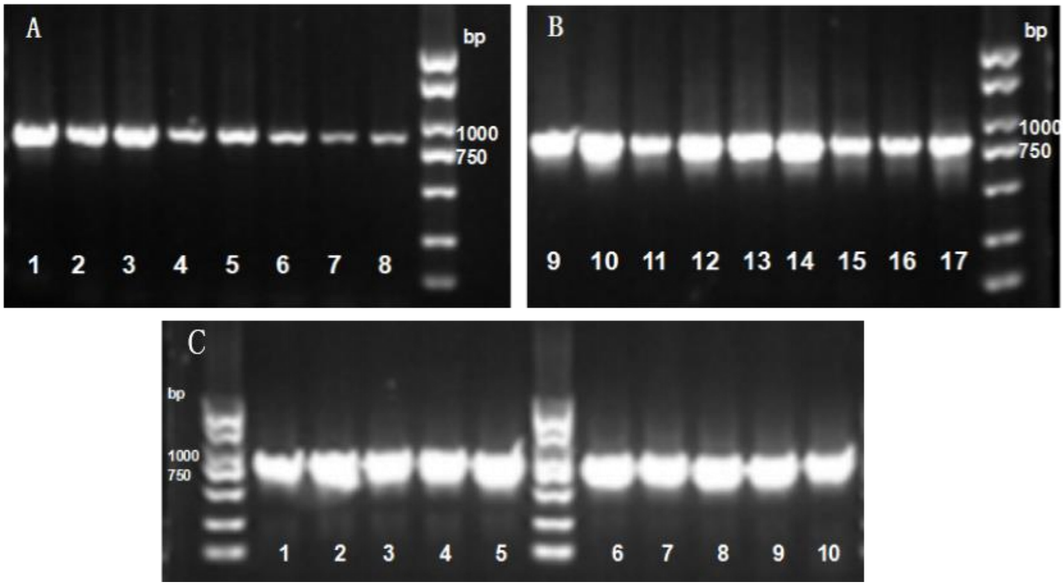


**Figure S1.** The detection of gel electrophoresis of *momL* fragment with series of epPCR condition. (A) The concentration gradient of $Mg^{2+}$. 1. 1mM; 2. 2mM; 3. 3mM; 4. 4mM; 5. 5mM; 6. 6mM; 7. 7mM; 8. 8mM. (B) The concentration gradient of $Mn^{2+}$. 9. 0mM; 10. 0.05mM; 11. 0.10mM; 12. 0.15mM; 13. 0.20mM; 14. 0.30mM; 15. 0.4mM; 16. 0.5mM; 17. 0.6mM. (C) The different concentration gradient test of $Mn^{2+}$ and $Mg^{2+}$. 1. $Mg^{2+}$ 1mM, $Mn^{2+}$ 0.00mM; 2. $Mg^{2+}$ 1mM, $Mn^{2+}$ 0.05mM; 3. $Mg^{2+}$ 1mM, $Mn^{2+}$ 0.10mM; 4. $Mg^{2+}$ 1mM, $Mn^{2+}$ 0.15mM; 5. $Mg^{2+}$ 1mM, $Mn^{2+}$ 0.20mM; 6. $Mg^{2+}$ 2mM, $Mn^{2+}$ 0.00mM; 7. $Mg^{2+}$ 2mM, $Mn^{2+}$ 0.05mM; 8. $Mg^{2+}$ 2mM, $Mn^{2+}$ 0.10mM; 9. $Mg^{2+}$ 2mM, $Mn^{2+}$ 0.15mM; 10. $Mg^{2+}$ 2mM, $Mn^{2+}$ 0.20mM.

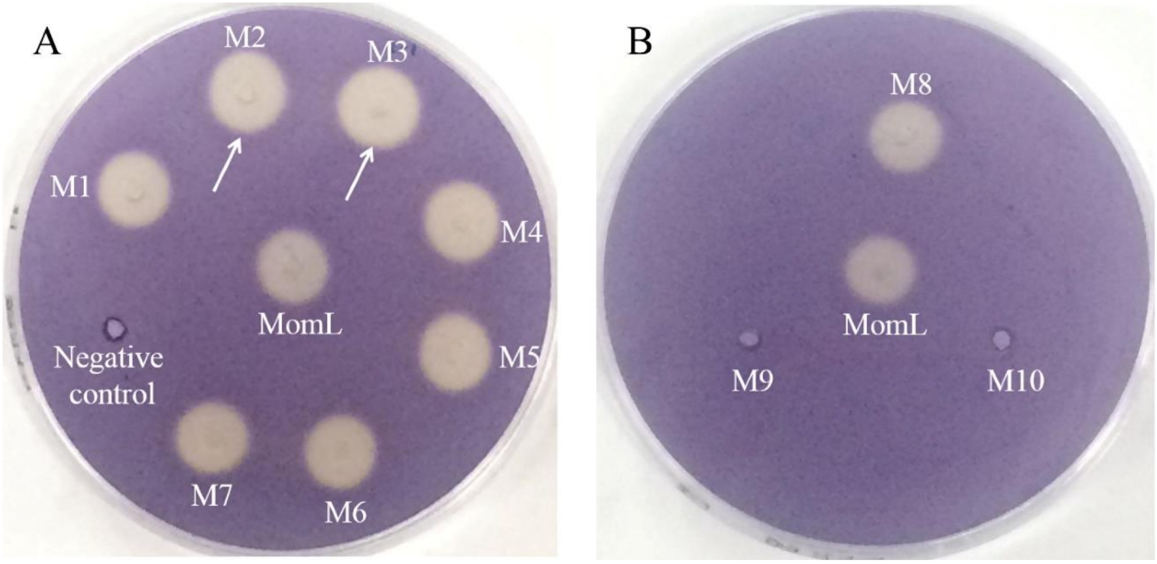


**Figure S2.** The protein activity test. In second round screening step, IPIG was not added to the CV026-loaded screening plate. White halos indicate that MomL and its mutants degraded C6-HSL. QQ ability of MomL and its mutants were determined due to halo diameter.

**Table S1.** Mutation sites and mutation primers of MomL. Primers with mutant sites were designed using the Exsite™ method. Mutagenic bases are underlined.

| protein | primer name | sequence | mutation site |
|---|---|---|---|
| MomL$_{N51Y}$ | N51Y-F | 5-GGAGGTACCGTATATGCCAATATGT-3 | Asn51→Tyr |
| | N51Y-R | 5- GCTAAAAGCATAGAGCTTTATCTCG-3 | |
| MomL $_{N179S}$ | N179S-F | 5-CCGGACATCTACAGTTCCATTAAAG-3 | Asn179→Ser |
| | N179S-R | 5-ATTGCTCTTTTGGTTGTCCTCGCTT-3 | |
| MomL $_{M228V}$ | M228V-F | 5-CCGTTGGTGCTTTCTGGGGAC-3 | Met228→Val |
| | M228V-R | 5-TCCGTGCTCAACCATATCCAA-3 | |
| MomL $_{K205E}$ | K205E-F | 5- GTAATGGAATTTATGCCAGGC-3 | Lys205→Glu |
| | K205E-R | 5- CACACTTCCATCCCCGAACAC-3 | |
| MomL $_{E238G}$ | E238G-F | 5-TACCATTTTTACGGGAACCGGGAGT-3 | Glu238→Gly |
| | E238G-R | 5-CATGTCCCCAGAAAGCATCAACGGT-3 | |
| MomL $_{L254R}$ | L254R-F | 5-AATTACGATGTGGCCCGCACCAAGA-3 | Leu254→Arg |
| | L254R-R | 5-AAAAATGGGCACTCTTCGGAACTCC-3 | |
| MomL $_{T84A}$ | T84A-F | 5-GTTCACCCCAGGGGCACTTTGATGT-3 | Thr84→Ala |
| | T84A-R | 5-GATGACGTAAAAAGCATCGGCAAAT-3 | |
| MomL $_{K82R}$ | K82R-F | 5-GTTCACCCCAGGGGCACTTTGATGT-3 | Lys82→Arg |
| | K82R-R | 5-GATGACGTAAAAAGCATCGGCAAAT-3 | |

**Table S2.** The mutation rate comparison of ep-PCR products with different conditions.

| PCR conditions | | no mutation | premature termination | number of mutated bases | | | | | | | | | | |
|---|---|---|---|---|---|---|---|---|---|---|---|---|---|---|
| $Mg^{2+}$ (mM) | $Mn^{2+}$ (mM) | | | 1 | 2 | 3 | 4 | 5 | 6 | 7 | 8 | 9 | 10 | 11 |
| 1.0 | 0.2 | 23% | 8% | 31% | 16% | 22% | - | - | - | - | - | - | - | - |
| 2.0 | 0.1 | 0 | 20% | 10% | 40% | - | 10% | 20% | - | - | - | - | - | - |
| 2.0 | 0.15 | 0 | - | - | 25% | 25% | - | 33% | 8% | 9% | - | - | - | - |
| 2.0 | 0.20 | 0 | 30% | - | - | 30% | - | - | - | 10% | 10% | 10% | - | 10% |
